# Genetically informed search for potential osteoarthritis drug targets across the proteome

**DOI:** 10.1016/j.ocarto.2026.100890

**Published:** 2026-10-24

**Authors:** Weijie Liu, Benjamin P. Zuckerman, Art Schuermans, Gisela Orozco, Michael C. Honigberg, John Bowes, Terence W. O'Neill, Sizheng Steven Zhao

**Affiliations:** a Centre for Musculoskeletal Research, Division of Musculoskeletal and Dermatological Science, School of Biological Sciences, Faculty of Biology, Medicine and Health, The University of Manchester, Manchester Academic Health Science Centre, Manchester, UK; b Centre for Rheumatic Diseases, King's College London, London, UK; c Faculty of Medicine, KU Leuven, Leuven, Belgium; d Program in Medical and Population Genetics, Broad Institute of MIT and Harvard, Cambridge, MA, USA; e Centre for Genomic Medicine, Massachusetts General Hospital, Boston, MA, USA; f Centre for Genetics and Genomics Versus Arthritis, Division of Musculoskeletal and Dermatological Sciences, School of Biological Sciences, Faculty of Biology, Medicine and Health, The University of Manchester, Manchester, UK; g NIHR Manchester Biomedical Research Centre, Manchester University NHS Foundation Trust, Manchester, UK; h Department of Medicine, Harvard Medical School, Boston, MA, USA

**Keywords:** Osteoarthritis, Mendelian randomisation, Proteomics, Drug targets, Genetic epidemiology

## Abstract

**Objective:**

Osteoarthritis (OA) is a leading cause of disability, yet no licensed therapies can prevent or slow its progression. We aimed to identify potential disease-modifying OA drug (DMOAD) targets by integrating genetic and differential protein abundance (DPA) evidence.

**Method:**

We evaluated genetically predicted perturbations of plasma protein levels using *cis*-protein quantitative trait loci (*cis*-pQTLs) across three European cohorts (UK Biobank Pharma Proteomics Project, deCODE, and Fenland) and outcome data from the Genetics of Osteoarthritis Consortium across 11 OA phenotypes. Following limit-of-detection and missingness filtering, DPA evaluated 2133 proteins in 7186 prevalent OA cases and 29,584 controls from UK Biobank. MR-prioritised proteins were further assessed using summary-data-based Mendelian randomisation (SMR) with OA joint-tissue eQTL data, alongside pathway, protein-interaction, and druggability analyses.

**Results:**

*Cis*-MR and colocalisation prioritised 81 proteins, of which 30 showed joint-tissue transcriptomic support in SMR analyses. DPA identified 1436 proteins associated with ≥1 OA phenotype, of which 1314 (91.5%) retained significance in sensitivity analyses. Seventeen proteins had matched MR and DPA support, including three Tier 1 druggable targets (DLK1, SOST, and TNFRSF9); seven of these had SMR support. EHBP1, LGALS3, and OGN showed concordant DPA and MR effects and cross-platform evidence. EHBP1, PCSK7 and ITIH4 emerged as under-recognised candidates. Pathway analyses recapitulated established OA processes, while drug enrichment identified hypothesis-generating compounds.

**Conclusion:**

Integration of *cis*-MR, colocalisation, DPA, and tissue-specific transcriptomic evidence prioritised several potential OA therapeutic targets. DLK1, LGALS3, OGN and EHBP1 emerged as high-priority candidates, while EHBP1, PCSK7 and ITIH4 represent under-recognised candidates warranting functional investigation.

## 1 Introduction

Osteoarthritis (OA) is a major cause of disability in middle-age and older adults [1]. The most frequently affected joints are the hip, knee, hand, foot and spine, although OA can affect any joint. It is estimated that symptomatic OA affects one in eight adults in the US [2]. Current management strategies have focused on reducing symptoms [3]. To date there are no licensed pharmacologic therapies that can prevent the onset or progression of OA.

Drug development in OA can be challenging, and clinical trial programmes have been largely unsuccessful to date. Most drug targets are proteins; therefore, one common approach to drug discovery is to perform hypothesis-free comparisons of circulating protein level between OA cases and controls [4], [5]. Observational proteomic associations may not reflect causation, correlated proteins can obscure biological signals, and existing proteome-wide OA studies remain limited by relatively small case numbers [6], [7], [8], [9].

Another increasingly popular approach is to use human genetic data to improve causal inference [10]. Studies have repeatedly shown that drugs supported by human genetic data are more than twice as likely to succeed [11]. Natural genetic variation in genes encoding protein drug targets can offer insights into the potential benefits - or risks - of targeting those proteins pharmacologically. Such Mendelian randomisation (MR) studies can add strength to observational analyses by explicitly seeking to estimate causation. Recent studies have identified numerous genetic variants associated with circulating protein levels (protein quantitative trait loci, pQTLs) using data from thousands of individuals [12], [13], [14]. Such variants can be used to evaluate the specific effects of genetically predicted levels of certain proteins on disease incidence [12], [15]. MR has been used for drug discovery in OA but mostly without replication across populations or proteomic technologies [6], [8], [9], [16]. MR studies, when presented alone, can also be problematic because, first, their primary inference pertains to OA prevention rather than modifying existing OA; second, effect sizes can be difficult to interpret as they reflect life-long rather than shorter-duration pharmacological interventions.

We address respective limitations of each drug discovery approach by combining case-control protein association and MR designs, in the largest sample size of either approach to date, to identify and prioritise potential OA therapeutic targets.

## 2 Methods

This study used UK Biobank (UKB), deCODE, and Fenland data. UKB recruited approximately 500,000 adults aged 40–69 years [17] during 2006–2010 [18]. Further details are provided in the Supplementary Methods.

### 2.1 Mendelian randomisation (MR) analysis

We used genetic association data for circulating protein levels from three independent sources: 1) UKB Pharma Proteomics Project (UKB-PPP) [14]: 2941 assays covering 2923 unique proteins measured with the Olink Explore 3072 platform in 54,219 participants (mean age 56.2 years, SD 8.2; 53.9% female). 2) deCODE (Iceland) [13]: 4907 assays of 4672 unique proteins using the SomaScan v4 platform (SomaLogic, Inc.) in 35,559 participants (mean age 55 years, SD 17; 57% female among those with plasma samples). 3) Fenland (UK) [12]: 4923 assays of 4665 unique proteins using SomaScan v4 in 10,708 participants (mean age 48.6 years, SD 7.5; 53.3% female). Only European ancestry data from these 3 data sources were used in the analysis.

We selected single-nucleotide variants as instrumental variables to proxy protein perturbation in each dataset. Because *cis*-MR restricts instrument selection to a biologically defined region around the encoding gene (±200 kb), we used P < 5 × 10^−6^ as the primary instrument-selection threshold; similar relaxed thresholds have been evaluated in *cis*-MR, although the optimal threshold is method- and context-dependent [19], [20]. *Cis*-SNVs with MAF >0.1% were clumped at r^2^ < 0.1 within a 10 Mb window using the 1000 Genomes Project Phase 3 v5a reference panel [21], to retain sufficient *cis*-instrument coverage, with residual correlation explicitly accounted for using the linkage disequilibrium (LD) correlation matrix [22] in multi-SNV MR analyses. Single-nucleotide variants within the Major Histocompatibility Complex region (chr 6: 26–34 Mb) were excluded. We prioritised *cis*-pQTLs because *trans*-pQTLs are more likely to be pleiotropic. To reduce bias from reverse causation (i.e., false positive associations due to OA affecting protein level), we performed Steiger filtering [23], that is, excluded pQTLs that explained greater variance in OA than protein level. F-statistics were calculated to assess potential weak instrument bias.

Outcome data were selected from the largest genome-wide association study (GWAS) Meta-Analysis of OA to date (Genetics of Osteoarthritis Consortium 2·0 [GO 2·0]), comprising approximately 1.9 million individuals (489,975 cases), of whom approximately 87.3% were of European ancestry [24]. The meta-analysis included 11 OA phenotypes: All OA, Knee and Hip OA, Hip OA, Knee OA, Total joint replacement (TJR), Total hip replacement, Total knee replacement, Spine OA, Hand OA, Finger OA and Thumb OA [24]. OA was defined using a combination of self-report, clinical diagnosis, International Classification of Diseases (ICD) codes, radiographic evidence and/or joint replacement, in each study within the meta-analysis as detailed in the original publication [24]. Controls were either OA-free or population-based, with some cohorts applying International Classification of Diseases code (e.g. rheumatoid arthritis and gout) or self-report exclusions.

MR analyses were conducted for each protein from each of three data sources versus each OA phenotype. Protein levels were analysed using standardised scores derived from the rank-based inverse normal transformation applied in the original pQTL studies (UKB-PPP, deCODE, and Fenland). Therefore, genetic association estimates for the exposures represent the per-allele change in protein abundance in standard deviation units rather than raw concentrations. OA was analysed as a binary trait (case vs control) in the corresponding GWAS datasets, and MR estimates are interpreted as the change in the odds of OA per one-SD increase in plasma protein level. In the MR analysis, we assumed that the *cis*-pQTL instruments were robustly associated with circulating protein levels (relevance), were not confounded by other factors (independence), and affected OA risk only through their impact on the corresponding protein (exclusion restriction). We used the Wald ratio method for single-SNV proteins, and fixed-effect inverse-variance weighted method for multi-SNV instruments incorporating LD structure to account for weak correlation. Benjamini-Hochberg false discovery rate (BH-FDR) correction was applied per OA phenotype, with global correction per proteomic data source as a sensitivity analysis. For MR associations that remained significant after multiple-testing correction, we performed Bayesian colocalisation analysis [25] to assess whether the genetic associations with protein abundance and OA were consistent with a shared causal variant rather than distinct causal variants within the same locus [25], [26]. Colocalisation was performed using all available variants within the *cis*-region rather than the LD-clumped instrument set used for MR. Default prior probabilities were used [26] and posterior probability of >80% was considered evidence of colocalisation. Proteins meeting both the MR significance and colocalisation criteria were considered MR-prioritised proteins. For proteins colocalised with ≥2 joint-specific OA phenotypes, MR instrument overlap was quantified using the Jaccard index (intersection/union of SNP sets), with ≥0.8 considered high overlap. Sensitivity analyses restricted instrument selection to genome-wide significant *cis*-pQTLs (P < 5 × 10^−8^), with more stringent LD pruning (r^2^ < 0.01). Finally, reverse MR used independent genome-wide significant OA instruments (P < 5 × 10^−8^; r^2^ < 0.001) that passed Steiger filtering [23].

We used summary-data-based Mendelian randomisation (SMR; version 1.4.2) with the HEIDI test to assess tissue-specific transcriptomic support for the 81 MR-prioritised proteins across OA phenotypes using cartilage, synovium, and infrapatellar fat pad eQTL datasets. Associations were considered supported at global BH-FDR ≤0.05 with HEIDI P ≥ 0.05. Full details of eQTL processing and multiple-testing correction are provided in the Supplementary Methods.

Analyses followed the STROBE-MR guidelines and were conducted in R (version 4.4.3) using ‘TwoSampleMR’ v0.6.9, ‘MendelianRandomization’ v0.10.0, and ‘coloc’ v5.2.3 [23], [25], [26], [27].

### 2.2 Differential protein abundance (DPA) analysis

Only UKB data were used in this analysis. Olink quantifies relative protein abundance on a log_2_ NPX scale, such that a 1-unit increase corresponds mathematically to a two-fold increase in relative protein abundance [14]. Proteins and participants with >30% missing protein measurements were excluded to minimise potential assay- and sample-quality issues.

We identified OA cases and controls among participants for whom Olink proteomic data were available. OA was defined as prevalent cases, that is, individuals with OA through self-report, ICD code [7], and/or primary care Read code, and/or OPCS code [28] before baseline blood sampling, while controls were participants without any recorded OA before baseline.

Linear regression was used with plasma protein level as the dependent variable and OA status as the main explanatory variable, adjusting for age, sex, and body mass index (BMI, kg/m^2^); sensitivity analyses additionally adjusted for the top three genetic principal components, smoking status (ever/never), and Townsend Deprivation Index.

Missing protein levels were imputed using the K-nearest neighbour method (KNN; k = 10). KNN imputation performance was assessed by randomly masking 2.32% of observed protein measurements, matching the overall missingness rate in the post-QC, pre-imputation protein matrix. This was repeated 10 times while retaining original missing values, and imputed values were compared with observed values at the masked entries. For BMI, values originally derived from measured height and weight were first replaced with impedance-based estimates where available. Remaining missing values for BMI, smoking status (ever/never), and Townsend Deprivation Index were then singly imputed using chained equations. The imputation model included age, sex, and the top 10 genetic principal components as auxiliary variables. BH-FDR correction was applied per OA phenotype.

### 2.3 Biological insights

To further prioritise and provide functional context for candidate proteins, we performed a series of analyses using proteins identified from MR and DPA analysis respectively.

#### 2.3.1 Over-representation analysis

The primary enrichment input comprised all 81 MR-prioritised proteins, irrespective of DPA support. Gene Ontology (GO) over-representation analysis was performed for Biological Process, Cellular Component, and Molecular Function categories [29]. Candidate proteins were also assessed using WikiPathways 2024 [30] and the Drug Signature Database [31] to identify enriched biological pathways and drug-associated gene sets, respectively. These enrichment analyses were exploratory and hypothesis-generating. To reduce enrichment bias, the background universe was restricted to all unique proteins measurable across the three proteomic GWAS datasets.

#### 2.3.2 Protein-protein interactions (PPI) network

We examined physical and functional PPI using STRING [32], and centrality measures were calculated with the CytoNCA app [33] in Cytoscape (v3·10·3).

#### 2.3.3 Druggability assessment

Candidate proteins were classified according to a published three-tier druggability framework [34], with Tier 1 representing targets of approved or clinical-stage drugs, Tier 2 comprising proteins related to established drug targets or with drug-like ligands, and Tier 3 comprising extracellular proteins and members of major druggable protein families. In addition, we performed a complementary druggability assessment using data from the Therapeutic Target Database [35] and the Open Targets Platform [36].

## 3 Results

Primary results from this study are summarised in Supplementary Table S1.

### 3.1 MR analysis

From the *cis*-MR analyses, we identified 81 unique proteins prioritised by *cis*-MR and colocalisation with at least one OA phenotype (Supplementary Table S2): 34 from UKB-PPP, 39 from deCODE, and 47 from Fenland (Supplementary Table S3). Across all OA phenotypes combined, 28 proteins-OA associations were replicated in at least one other data source with 11 (including PCSK7) replicated in all three. Nine from UKB-PPP and 2 from each of deCODE and Fenland could not be replicated due to their unique presence in each data source (Supplementary Fig. S1; Supplementary Table S4). Forty proteins showed support across at least one additional OA phenotype, while no proteins were supported across all 11 OA phenotypes (Supplementary Table S5 and Supplementary Fig. S2). Fig. 1 shows Miami plots for all-OA; phenotype-specific plots are shown in Supplementary Fig. S3–5. Global BH-FDR sensitivity analysis retained all but two prioritised MR associations (cartilage oligomeric matrix protein and sushi domain-containing protein 2), without materially altering the primary findings (Supplementary Table S6).

**Fig. 1:**
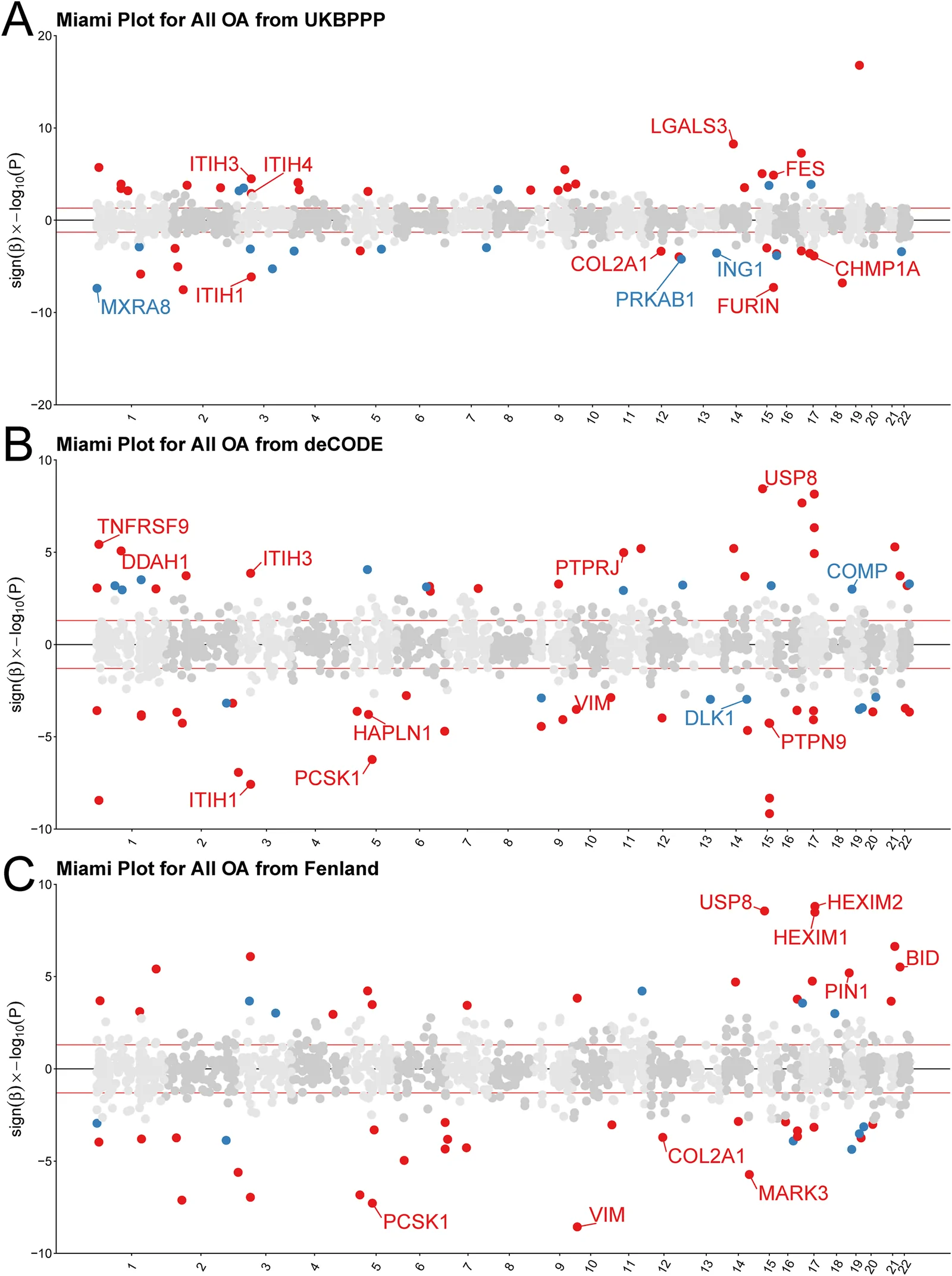
Miami plot showing the results of all OA trait from MR analyses based on UKB-PPP, deCODE and Fenland (Fig. 1A–C). The x-axis represents the chromosomal position of each protein's encoding gene, while the y-axis shows the sign(β) multiplied by the negative log_10_-transformed p-value (after B–H procedure), allowing visualization of effect directions. Positive values indicate that genetically predicted higher protein abundance is associated with increased OA risk, whereas negative values indicate an association with decreased OA risk. The two red horizontal lines mark the significance threshold (p = 0.05). Proteins in blue show MR evidence only in the current OA phenotype, whereas those in red are supported by MR evidence in two or more OA phenotypes. Proteins that meet colocalisation criteria (PPH_4_ > 0.8) are labelled with their protein names. OA, osteoarthritis; MR, mendelian randomisation; UKB-PPP, UK biobank Pharma Proteomics Project; B–H, Benjamini-Hochberg; PPH_4_, posterior probability of hypothesis 4.

Among the candidate proteins, protein polybromo-1 (PBRM1) showed the strongest association (by p-value) with knee and hip OA (Wald ratio: OR = 1.6, 95% confidence interval:1.4–1.8; *P* < 4 × 10^−16^), followed by matrix gla protein (MGP) with finger OA (OR = 0.8, 95% CI: 0.7–0.8; *P* < 1 × 10^−15^), and inter-alpha-trypsin inhibitor heavy chain H3 (ITIH3) with Total joint replacement (TJR) (OR = 1.2, 95% CI: 1.1–1.2; *P* < 1 × 10^−14^; Supplementary Table S1). Both MGP and ITIH3 showed additional support across pQTL resources and/or OA phenotype. Among 40 proteins colocalised with ≥2 OA phenotypes, 31 (78%) showed high instrument overlap (Jaccard index ≥0.8), suggesting largely shared genetic instruments across joints. DDAH1, DLK1, HHIP, ITIH3, and tumour necrosis factor receptor superfamily member 9 showed comparatively lower instrument overlap across joints (Supplementary Table S7).

All instruments had F-statistics >20, and reverse MR provided no evidence of reverse causation (Supplementary Tables S8–10). Under the more stringent instrument-selection strategy (P < 5 × 10^−8^; r^2^ < 0.01), 70 of the 81 (86.4%) MR-prioritised proteins retained significant MR support; 66 of the original 81 (81.5%) retained support in source-phenotype pairs with primary colocalisation evidence. Effect directions were concordant among associations significant under both instrument-selection strategies, although the overall set of significant associations differed (Supplementary Tables S1–S2).

Among 17,569 unique SNP-OA associations with available heterogeneity data from colocalisation-supported findings, there was little evidence of between-study heterogeneity: 746 (4.2%) showed nominal heterogeneity (P-heterogeneity <0.05), and 166 (0.9%) had I^2^ ≥ 50% (Supplementary Table S8).

Among the 81 MR-prioritised proteins, 30 proteins showed tissue-specific transcriptomic support, defined as global BH-FDR ≤0.05 in SMR with a finite HEIDI P value ≥ 0.05 (Supplementary Table S11).

### 3.2 DPA analysis

The DPA cohort included 7186 OA cases and 29,584 controls; cases were older (61.0 vs 55.8 years), more often female (60.5% vs 51.4%), and had higher BMI (29.0 vs 27.1 kg/m^2^) (Supplementary Table S12). Proteins with >30% of samples below the limit of detection, and participants and proteins with >30% missing protein measurements, were excluded, leaving 2133 proteins for DPA. Among retained proteins, 2.32% of measurements were missing and imputed using K-nearest neighbours (KNN; k = 10; median protein-level missingness, 2.00%; IQR, 1.25–3.46%). In 10 masking-based validation repetitions, 2.32% of observed measurements were randomly masked, with mean performance showing moderate agreement between imputed and observed values (Pearson r = 0.676; Spearman r = 0.607; RMSE = 0.512 NPX units; MAE = 0.335 NPX units). We identified 1436 unique proteins associated with at least one OA phenotype after multiple testing correction. The number of significant proteins varied by phenotype, from 1124 for all OA to two for the thumb OA. Results for each OA phenotype are shown in Supplementary Table S13.

Among these results, collagen alpha-1(IX) chain (COL9A1) showed the most significant signal with all OA, followed by kallikrein-4 (KLK4) and CUB domain-containing protein 1 with TJR. Fig. 2 summarises results for all OA in a volcano plot, with corresponding volcano plots for the other ten OA phenotypes shown in Supplementary Fig. S6. Most proteins (1314/1436; 91.5%) retained significant DPA support for at least one matched OA phenotype in sensitivity analysis adjusting for additional covariates (Supplementary Table S14). Notably, the association signals observed for COL9A1 and CRTAC1 are consistent with previous reports [37], [38], [39], [40].

**Fig. 2:**
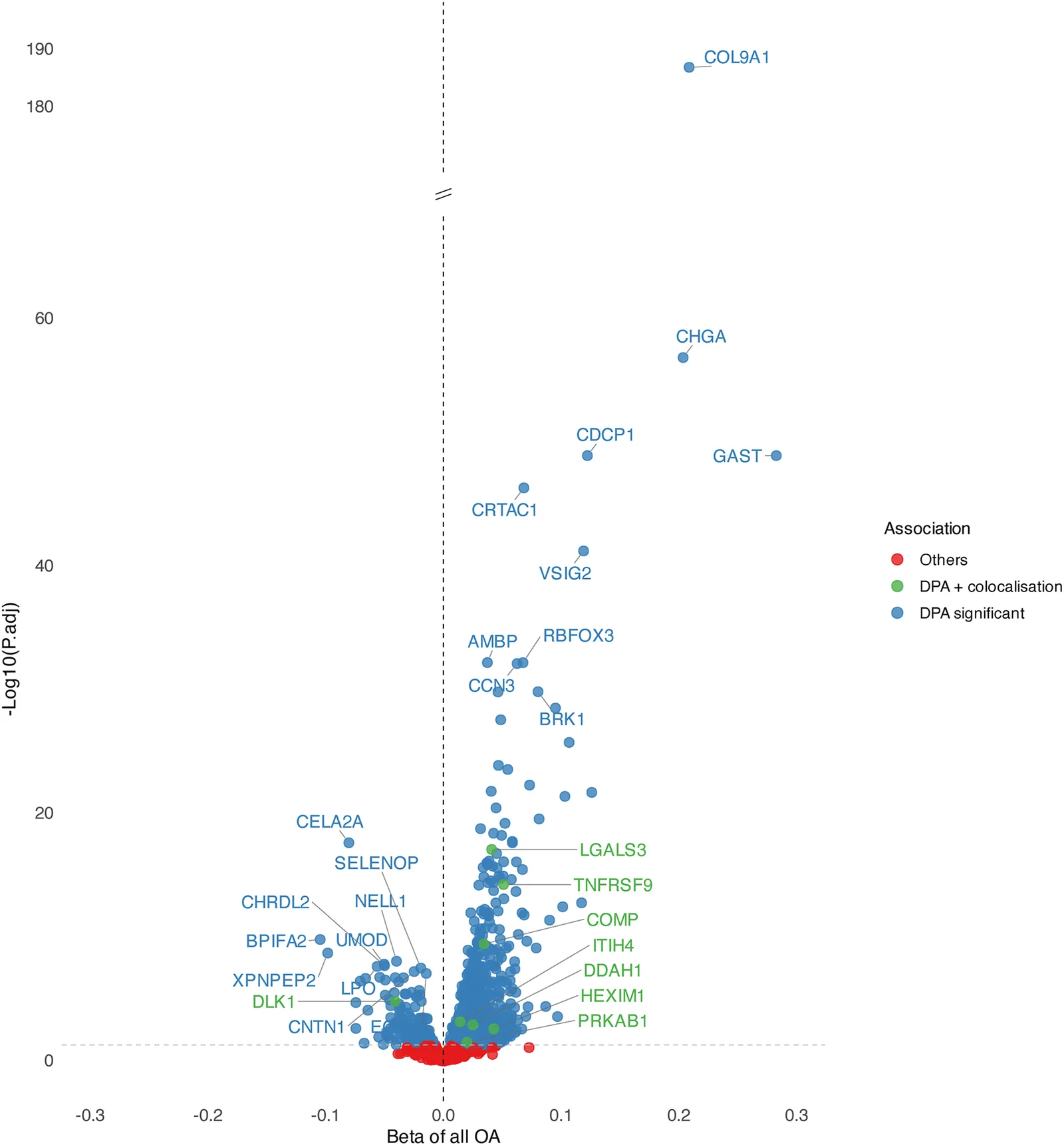
Volcano plot of the DPA analysis for all OA. The x-axis shows regression coefficients representing adjusted differences in NPX between OA cases and controls; positive values indicate higher protein abundance in OA cases and negative values indicate lower abundance. The y-axis indicates the -log 10 of the adjusted p-values from linear regression. Proteins overlapping between DPA and MR-prioritised proteins across three proteomic datasets with matched OA types are shown in green. Among the most significant proteins based on p-values, the top 10 on each side of the beta distribution are labelled. Other proteins are shown in red. DPA, differential protein abundance; OA, osteoarthritis; OR, odds ratio.

### 3.3 Overlap between DPA and MR analysis

When comparing matched OA phenotypes, 17 proteins were supported by both *cis*-MR with colocalisation and DPA, including three Tier 1 druggable targets (Table 1). Protein delta homolog 1 (DLK1) was associated with reduced OA risk in MR and lower circulating levels in knee and hip OA and knee OA. Sclerostin (SOST) was associated with reduced OA risk in MR but higher circulating levels in TKR, whereas tumour necrosis factor receptor superfamily member 9 was associated with increased OA risk in MR and higher circulating levels in all OA, knee and hip OA, and hip OA.

**Table 1:** Summary of MR-Prioritised proteins with tier 1 druggability support^a^.

Protein	Available data sources	Data sources with MR signals	OA phenotypes with MR signals	Support from both DPA and MR^b^	PPI center^c^
DLK1	All 3 data sources	All 3 data sources	Alloa; KNEEHIP; knee; TKR	Yes	No
DPEP1	All 3 data sources	deCODE; UKB-PPP	HIP; KNEEHIP; knee; TJR; TKR	No	No
PDGFRA	All 3 data sources	UKB-PPP; fenland	THR	No	No
SOD3	All 3 data sources	UKB-PPP; fenland	HIP; KNEEHIP; THR; TJR	No	No
VIM	All 3 data sources	deCODE; fenland	Alloa; HIP; KNEEHIP; TJR	No	No
CSK	deCODE; Fenland	deCODE	TKR	NA	No
CXCL10	All 3 data sources	Fenland	HAND	No	Yes
DDR2	deCODE; Fenland	Fenland	TKR	NA	No
FGFR3	deCODE; Fenland	Fenland	KNEEHIP; THR; TJR	NA	Yes
FGFR4	All 3 data sources	UKB-PPP	Knee; TKR	No	No
MAPK3	deCODE; Fenland	deCODE	KNEE	NA	Yes
MAPK9	All 3 data sources	deCODE	HAND	No	No
MARK3	deCODE; Fenland	Fenland	Alloa; KNEEHIP; knee; TJR; TKR	NA	No
PRKD2	UKB-PPP	UKB-PPP	HIP	No	No
SOST	All 3 data sources	Fenland	TKR	Yes	Yes
TNFRSF9	All 3 data sources	deCODE	Alloa; HIP; KNEEHIP; THR; TJR	Yes	No
ULK3	deCODE; Fenland	deCODE	TKR	NA	No

MR-prioritised proteins: proteins meeting both MR significance and colocalisation criteria; MR: Mendelian randomisation; All 3 data sources: UK Biobank-Pharma Proteomics Project (UKB-PPP), deCODE, and Fenland; OA: osteoarthritis; UKB-PPP: UK Biobank Pharma Proteomics Project; TJR: total joint replacement; TKR: total knee replacement; THR: total hip replacement; KNEEHIP: knee and hip OA; NA: not applicable if protein does not exist in UKB-PPP; PPI: protein-protein interaction; DPA: differential protein abundance.

a Tier 1: targets of approved drugs or drugs in clinical development.

b Support from both DPA and MR; same OA phenotype though could be different MR data source.

c PPI centres were defined according to the eight centrality metrics (see supplementary methods).

Of 17 proteins, seven had additional SMR support. And of these 7, only EH domain-binding protein 1 (EHBP1), galectin-3 (LGALS3) and mimecan (OGN) showed concordant DPA and MR findings and cross-platform evidence. EHBP1 (knee and hip), LGALS3 (all OA, knee, hip, knee and hip, THR and TKR) and OGN (TKR and knee) increased OA risk in MR and were also elevated in individuals with OA.

### 3.4 Biological insights

In the GO over-representation analysis of 81 MR-prioritised proteins, endochondral bone growth, collagen-containing extracellular matrix, and glycosaminoglycan binding were significantly enriched terms in BP, CC, and MF, respectively (Fig. 3; Supplementary Table S15). Among the WikiPathways analysed, Osteoarthritic Chondrocyte Hypertrophy was the only significantly over-represented pathway, while NVP-TAE684 and HG-9-91-01 showed the strongest over-representation in Drug Signature Database (Fig. 3).

**Fig. 3:**
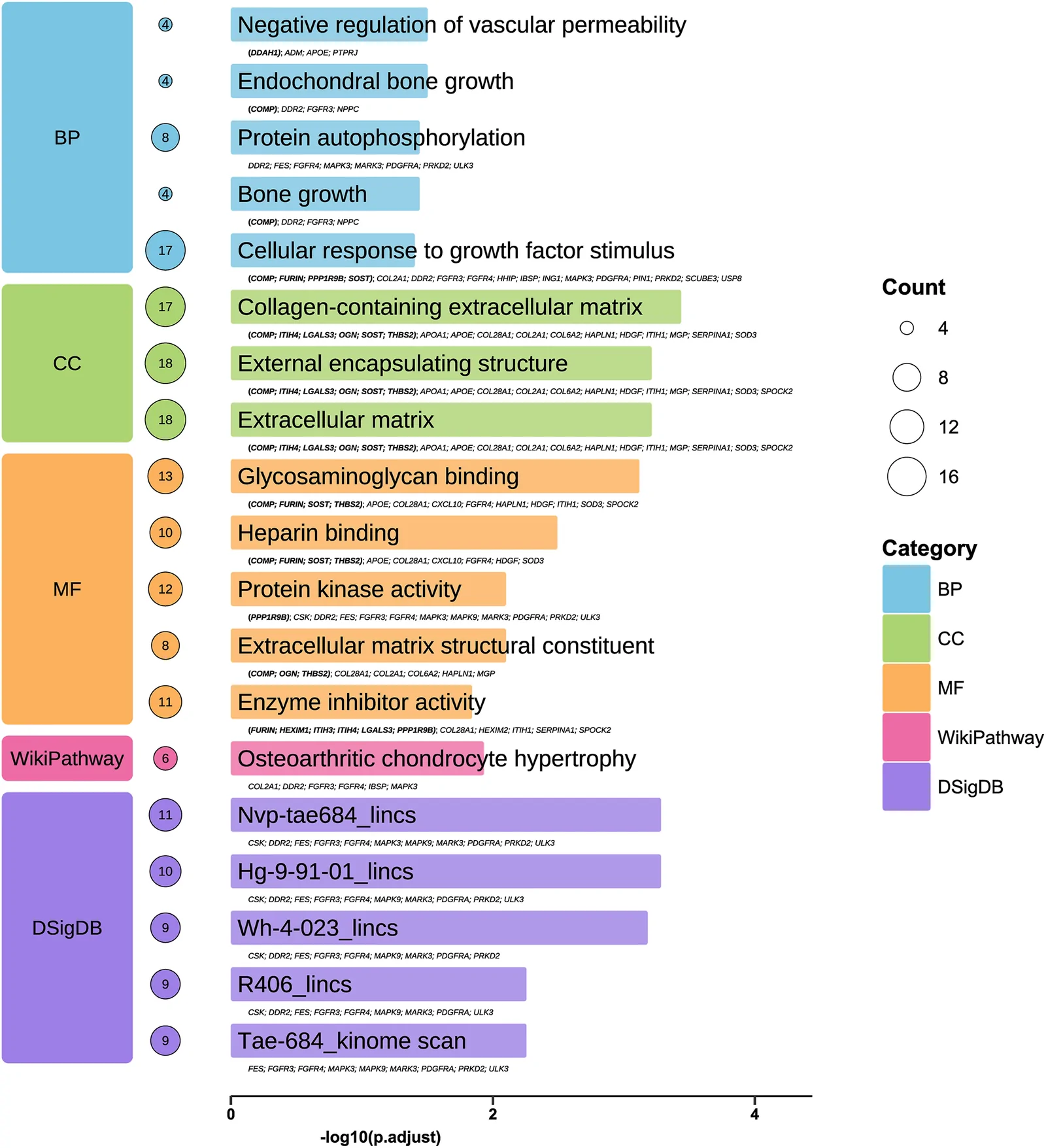
Top five over-representation analysis results based on 81 MR-prioritised proteins, using GO, WikiPathway, and DsigDB. The GO consists of BP, CC, and MF. For each pathway or term, the associated proteins are listed, with those supported by both DPA and MR highlighted in bold. Circle sizes represent the number of proteins enriched in each pathway or term. GO, Gene Ontology; DsigDB, Drug Signature Database; BP, biological process; CC, cellular component; MF, molecular function; OA, osteoarthritis; DPA, differential protein abundance; MR, mendelian randomisation.

The results using 1436 proteins with DPA evidence and using 34 MR-prioritised proteins from the UKB-PPP are presented in Supplementary Fig. S7 and Supplementary Fig. S8 for reference.

PPI network analysis was performed using the 81 MR-prioritised proteins (Fig. 4). The PPI results showed 79 nodes and 98 edges (expected number of edges: 51; *P* < 0.001), with proteins such as MAPK3 exhibiting the highest degree (degree = 13), suggesting a network hub. Fourteen proteins, including FGFR3 and ITIH4, were identified as central proteins using median-based filtering (Supplementary Methods). Betweenness centrality values from main PPI results suggest that MAPK3 also acts as a key connector in the network (Supplementary Table S16).

**Fig. 4:**
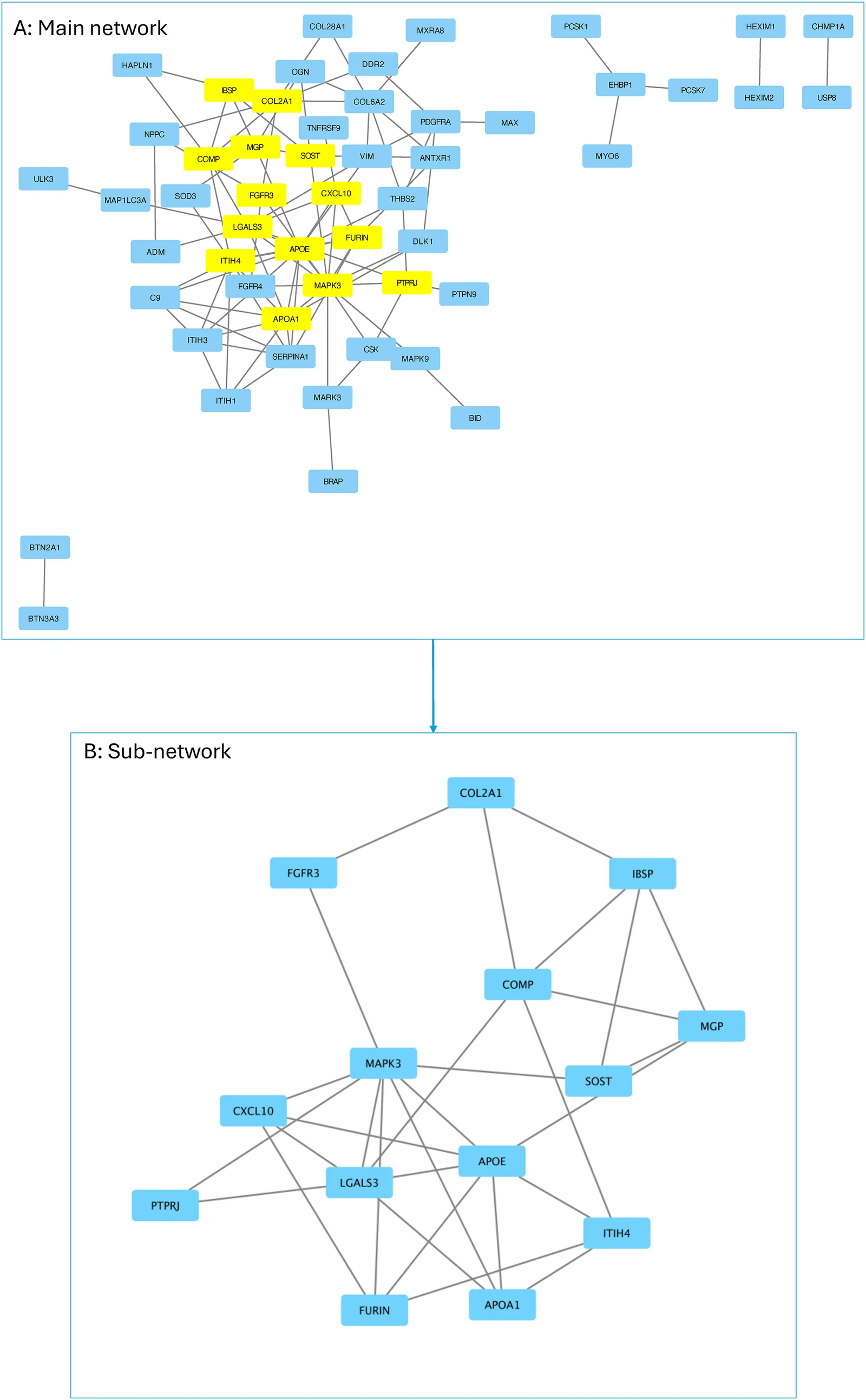
PPI network. Fig. 4A: PPI main network; proteins highlighted in yellow represent central proteins which are defined by eight centrality metrics (see supplementary methods); Fig. 4B: PPI sub-network constructed using the central proteins (as highlighted in Fig. 4a). Results generated by using 81 MR-prioritised proteins in the STRING database. PPI, protein-protein interaction; STRING, Search Tool for the Retrieval of Interacting Genes/Proteins.

Of the 81 MR-prioritised proteins, 10 replicated previously reported findings (including ART4 [6], [7], MGP [6], [9], DPEP1 [6], ITIH1 [6], [8], [9], HHIP [6], [9], MYORG [6], MAPK3 [8], LGALS3 [8], DNAJB12 [8], [9], USP8 [8], [9]). No association was observed, however, with ECM1 [6], SEMA3G [6], CDH6 [6], GZMK [8], OMG [8], IL12B [9] and RGMB [9] which have been previously reported. Of the 81 MR-prioritised proteins, 51 (63.0%) are considered druggable gene targets (Tier 1–3; including PCSK7 and ITIH4) (Supplementary Table S17). Among these, 17 proteins (Table 1) correspond to druggable genes that are either targets of approved drugs or targets of drugs currently in clinical development (Tier 1). 8 proteins (e.g. LGALS3) are further identified as Tier 1 druggable targets in either Target Database or Open Targets Platform (Supplementary Table S2).

## 4 Discussion

We integrated genetic evidence across three proteomic resources to prioritise potential therapeutic targets, with DPA and tissue-specific eQTL analyses providing complementary evidence. Compared with previous proteome-wide MR studies, we expanded pQTL coverage (3090 vs 1558 proteins [6]), leveraged the larger GO 2.0 GWAS (∼1.9 million vs ∼826,000 participants), and incorporated cross-platform (SomaScan and Olink) evidence [7], [24]. DLK1 emerged as a leading candidate, supported by multiple lines of evidence and also highlighted in the GO 2·0 study [24]. Our findings both reinforce established OA targets and prioritise under-recognised candidates for further investigation.

Among the 81 MR-prioritised proteins, PBRM1, MGP and ITIH3 showed the strongest primary MR associations, although PBRM1 was not retained under the stringent instrument-selection sensitivity analysis. Genetically predicted PBRM1 was associated with an increased risk of knee and hip OA in Fenland. PBRM1 lies within the 3p21 OA risk locus and has been implicated through cartilage methylation and chondrocyte splicing-QTL analyses [41], [42]; however, GNL3 has been prioritised as an effector gene at this locus [43]. Thus, our findings provide additional protein-level evidence for PBRM1 rather than establishing it as a novel OA target. MGP, associated with reduced finger OA risk, is an established OA susceptibility gene implicated in matrix mineralisation [44], with supporting evidence from previous pQTL-MR and GO 1.0 studies [6], [7]. ITIH3, which was associated with an increased risk of TJR in UKB-PPP and showed support across several OA phenotypes, has been implicated in other joint diseases [45] but not OA, suggesting a potential new therapeutic direction.

Among MR-prioritised targets with limited prior OA evidence, EHBP1 showed convergent support across DPA, MR, SMR and cross-platform analyses, whereas PCSK7 replicated across all three proteomic sources and was supported by SMR; and ITIH4 emerged as a central PPI hub with SMR support. EHBP1 regulates endocytic trafficking and links endosomal membranes to the actin cytoskeleton through interactions with Eps15-homology domain-containing proteins 1/2 proteins and Rab8/Rab 10 [46], suggesting that membrane trafficking and cytoskeletal regulation may represent plausible mechanisms linking EHBP1 to OA. PCSK7 encodes a proprotein convertase that processes multiple precursors involved in metabolic and inflammatory signalling and has been implicated in lipid metabolism and macrophage activation [47], supporting a potential metabolic/inflammatory OA mechanism. ITIH4 is an acute-phase protein within the inter-α-trypsin inhibitor family involved in protease regulation and extracellular matrix stabilisation [45], suggesting a possible link between inflammation, protease activity and matrix-related OA pathways. These findings should be considered hypothesis-generating pending functional validation.

Among the 7 proteins supported by both DPA and SMR, only LGALS3, OGN and EHBP1 (discussed above) showed concordant DPA and MR effects, survived both global BH-FDR correction and stringent MR sensitivity analysis, and had cross-platform support. LGALS3 encodes galectin-3, which is increased in OA synovium and can promote inflammatory and matrix-degradative responses involving Akt and NF-κB signalling [48]. In our study, LGALS3 levels were higher in OA cases, with concordant risk-increasing associations in both MR and SMR analyses. The study providing the primary OA joint-tissue eQTL resource used in our SMR analysis also prioritised LGALS3 as an OA effector gene, with higher expression in both low- and high-grade cartilage genetically associated with increased OA risk [49]. OGN, an extracellular matrix proteoglycan reported to be upregulated in OA cartilage [50], [51], was similarly elevated in OA cases, with genetically predicted higher levels associated with increased OA risk. Together, these findings support potential pathogenic roles for LGALS3 and OGN in OA, although their therapeutic relevance requires direct validation. In contrast, DLK1 was the only Tier 1 druggable target supported by DPA and cross-platform validation, but lacked SMR evidence. DLK1, a Notch/Delta family protein involved in bone remodelling [52], showed protective effects across multiple OA phenotypes and lower plasma levels in OA cases. Supporting a role for DLK1 in OA joint tissues, Hatzikotoulas et al. identified enrichment of *DLK1*-expressing chondrocytes across OA phenotypes [24]. Although enhanced *DLK1* expression has promoted regeneration in myocardium [53], whether activation has therapeutic benefit in OA requires direct validation.

Over-representation analysis implicated established OA processes, including endochondral bone growth, extracellular matrix organisation, glycosaminoglycan binding, and chondrocyte hypertrophy [54], [55], [56], [57], [58], supporting biological coherence rather than novel mechanisms. Negative regulation of vascular permeability was also enriched, suggesting a potential vascular component that has received less attention in OA. Drug enrichment identified NVP-TAE684, an ALK inhibitor that can suppress downstream ERK signalling, and R406, a compound with efficacy in rheumatoid arthritis [59], as hypothesis-generating candidates. The prioritisation also showed face validity against existing therapeutic evidence. Romosozumab targets SOST [60], whereas our MR analyses showed that genetically predicted higher SOST was associated with lower Total knee replacement risk, highlighting potential differences between lifelong genetic exposure and pharmacological inhibition. Similarly, sprifermin activates FGF receptors including FGFR3 and has demonstrated structural effects on cartilage [61], consistent with our finding that genetically predicted higher FGFR3 was associated with lower risks of knee/hip OA and joint replacement.

A major strength of this study is the integration of three large proteomic datasets (UKB-PPP, deCODE, and Fenland) with UKB case-control data. Several limitations should be considered. First, circulating protein levels may not fully reflect tissue-specific abundance within the joint, and single-time-point measurements cannot capture temporal changes. Second, the small sample sizes for some OA phenotypes, particularly hand, finger, and thumb OA, may have reduced statistical power. Third, the lack of sex-stratified data precluded assessment of sex-specific effects. Fourth, DPA could not be replicated in deCODE or Fenland because only summary-level data were available. Fifth, the primary *cis*-MR analysis used a relaxed instrument-selection threshold (P < 5 × 10^−6^), which may be more susceptible to weak-instrument and winner's-curse bias than genome-wide significant instrument selection. However, most MR-prioritised proteins retained support under the stringent P < 5 × 10^−8^ sensitivity analysis, with concordant effect directions among associations significant under both specifications. Findings not retained under the stringent specification should be interpreted with caution. Besides, participants overlap between the OA GWAS and pQTL datasets could not be excluded and may have introduced bias, although strong instruments may reduce, but not eliminate, this concern. Sixth, the restriction to European ancestry may limit generalizability to other populations. Seventh, several OA phenotypes shared cases and genetic architecture; therefore, cross-phenotype findings should not be considered independent replication. Finally, primary colocalisation analysis assumed a single causal variant; multi-signal approaches such as SuSiE-coloc may better resolve loci with multiple causal signals.

Integration of *cis*-MR and colocalisation across three proteomic resources prioritised several potential OA therapeutic targets, with DPA and tissue-specific SMR support providing complementary evidence. DLK1, LGALS3, OGN and EHBP1 - emerged as high-priority targets, with additional tissue-specific SMR support for OGN. Pathway analyses recapitulated established OA processes, including chondrocyte hypertrophy. Under-recognised candidates such as EHBP1, PCSK7 and ITIH4 warrant further wet-lab investigation, with PCSK7 and ITIH4 also supported by tissue-specific SMR analyses. Drug enrichment results (e.g., NVP-TAE684, R406) provide hypotheses for further investigation.

## Ethics approval

Use of UKB data was approved by the North West Multi-center Research Ethics Committee. Analyses were conducted under application no. 72723. Ethical approval was not required for analysis using summary-level data.

## Contributors

All authors made substantial contributions to: (1) the conception and design of the study, acquisition of data, or analysis and interpretation of data; (2) drafting the article or revising it critically for important intellectual content; and (3) final approval of the version to be submitted. WL takes overall responsibility for the integrity of the work, from inception to the final article. SSZ has directly accessed and verified the underlying data reported in the manuscript.

## Data sharing statement

UKB data are available to all bona fide researchers for use in health-related research that is in the public interest. The application procedure is described at http://www.ukbiobank.ac.uk/. Summary-level protein GWAS data (UKBPPP, deCODE, Fenland) and OA GWAS data (GO 2·0 consortium) are publicly available.

## Declaration of generative AI in scientific writing

During the preparation of this work, the authors used ChatGPT (OpenAI) to improve the language and readability of the manuscript. After using this tool, the authors reviewed and edited the content as needed and take full responsibility for the content of the publication.

## Funding

WL is supported by the Guangzhou Elite Project (project no. JY202314). SSZ is supported by 10.13039/501100000770The University of Manchester Dean's Prize and an Arthritis 10.13039/100007472UK Career Development Fellowship (grant no. 23258).

## Declaration of interests

M.C.H. reports site principal investigator work for Novartis and research support from Genentech. All other authors report no relevant competing interests.
