# Supplementary material for: Genetically informed search for potential osteoarthritis drug targets across the proteome
Source: Osteoarthr Cartil Open. 2026 Oct 24;8(4):100890. doi: 10.1016/j.ocarto.2026.100890 (PMC13757520; doi:10.1016/j.ocarto.2026.100890)
Supplement: Supplementary file 1 [file mmc1.docx]

**Supplementary figures for: Genetically informed search for potential osteoarthritis drug targets across the proteome**

Weijie Liu^1^, Ben P. Zuckerman^1,2^, Art Schuermans^3,4,5^, Gisela Orozco^6,7^, Michael C. Honigberg^4,5,8^, John Bowes^6,7^, Terence W O’Neill^1,7^*, Sizheng Steven Zhao^1,7^*

Author affiliations:

1.	Centre for Musculoskeletal Research, Division of Musculoskeletal and Dermatological Science, School of Biological Sciences, Faculty of Biological Medicine and Health, The University of Manchester, Manchester Academic Health Science Centre, Manchester, UK

2.	Centre for Rheumatic Diseases, King’s College London, London, UK

3.	Faculty of Medicine, KU Leuven, Leuven, Belgium

4.	Program in Medical and Population Genetics, Broad Institute of MIT and Harvard, Cambridge, MA, USA

5.	Centre for Genomic Medicine, Massachusetts General Hospital, Boston, MA, USA

6.	Centre for Genetics and Genomics Versus Arthritis, Division of Musculoskeletal and Dermatological Sciences, School of Biological Sciences, Faculty of Biology, Medicine and Health, The University of Manchester, Manchester, UK

7.	NIHR Manchester Biomedical Research Centre, Manchester University NHS Foundation Trust, Manchester, UK

8.	Department of Medicine, Harvard Medical School, Boston, MA, USA

*Contributed equally

Correspondence to Weijie Liu

Email: [weijie.liu-2@postgrad.manchester.ac.uk](mailto:weijie.liu-2@postgrad.manchester.ac.uk)

Phone: +44 07419204929

**Supplementary Fig. 1.** Venn diagram illustrating the overlap of MR results across the three cohorts. Numbers indicate proteins unique to each cohort or shared between cohorts. MR, mendelian randomisation; UKB-PPP, UK Biobank Pharma Proteomics Project.

**Supplementary Fig. 2.** Heatmap of pairwise overlaps in MR significant proteins across 11 OA phenotypes. The numbers within the cells indicate the number of proteins shared between each pair of OA types, with color intensity reflecting the degree of overlap. MR, mendelian randomisation; OA, osteoarthritis; TJR, total joint replacement; THR, total hip replacement; TKR, total knee replacement.

**Supplementary Fig. S3.** Miami plots of MR results across 10 OA (Supplementary Fig. S3A-S3J) phenotype in the UKB-PPP data source. The x-axis shows the chromosomal position of each protein’s encoding gene, and the y-axis represents the signed(β) multiplied by the negative log10-transformed p-value after Benjamini–Hochberg correction, allowing visualization of effect direction. The red horizontal lines indicate the nominal significance threshold (p = 0.05). Proteins highlighted in blue indicate MR associations specific to the current OA phenotype in current data sources, while those in red are supported by MR evidence in two or more phenotype. Proteins fulfilling colocalisation criteria (PPH_4_ > 0.8) are labeled. MR, mendelian randomisation; UKB-PPP, UK Biobank Pharma Proteomics Project; OA, osteoarthritis; PPH_4_, posterior probability of hypothesis 4; TJR, total joint replacement; THR, total hip replacement; TKR, total knee replacement.

**Supplementary Fig. S4.** Miami plots of MR results across 10 OA phenotypes (Supplementary Fig. S4A-S4J) in the deCODE data source. The x-axis shows the chromosomal position of each protein’s encoding gene, and the y-axis represents the signed(β) multiplied by the negative log10-transformed p-value after Benjamini–Hochberg correction, allowing visualization of effect direction. The red horizontal lines indicate the nominal significance threshold (p = 0.05). Proteins highlighted in blue indicate MR associations specific to the current OA phenotype in current data sources, while those in red are supported by MR evidence in two or more phenotype. Proteins fulfilling colocalisation criteria (PPH_4_ > 0.8) are labeled. MR, mendelian randomisation; UKB-PPP, UK Biobank Pharma Proteomics Project; OA, osteoarthritis; PPH_4_, posterior probability of hypothesis 4; TJR, total joint replacement; THR, total hip replacement; TKR, total knee replacement.

**Supplementary Fig. S5.** Miami plots of MR results across 10 OA phenotypes (Supplementary Fig. S5A-S5J) in the Fenland data source. The x-axis shows the chromosomal position of each protein’s encoding gene, and the y-axis represents the signed(β) multiplied by the negative log10-transformed p-value after Benjamini–Hochberg correction, allowing visualization of effect direction. The red horizontal lines indicate the nominal significance threshold (p = 0.05). Proteins highlighted in blue indicate MR associations specific to the current OA phenotype in current data sources, while those in red are supported by MR evidence in two or more phenotype. Proteins fulfilling colocalisation criteria (PPH_4_ > 0.8) are labeled. MR, mendelian randomisation; UKB-PPP, UK Biobank Pharma Proteomics Project; OA, osteoarthritis; PPH_4_, posterior probability of hypothesis 4; TJR, total joint replacement; THR, total hip replacement; TKR, total knee replacement.

**Supplementary Fig. S6.** Volcano plots of DPA results across 10 OA phenotypes (Supplementary Fig. S6A-S6J). The x-axes show beta coefficients representing the adjusted difference in protein abundance between cases and controls for the indicated OA phenotype, and the y-axes show –log10 of the adjusted p-values from linear regression. Proteins overlapping between DPA and MR analyses across three proteomic datasets with matched OA types are shown in green. The top 10 most significant proteins on each side of the beta distribution are labelled. Other proteins are displayed in red. DPA, differential protein abundance; OA, osteoarthritis; MDD, minimum detectable difference; MR, mendelian randomisation; TJR, total joint replacement; THR, total hip replacement; TKR, total knee replacement.

**Supplementary Fig. S7.** Top five over-representation analysis results based on 605 significant proteins in DPA analysis, using GO, WikiPathway, and DsigDB databases. Categories include BP, CC, MF, and DsigDB. Circle sizes represent the number of proteins enriched in each pathway or term. None of the pathways or terms reached statistical significance. DPA, differential protein abundance; GO, Gene Ontology; DsigDB, Drug Signature Database; BP, biological process; CC, cellular component; MF, molecular function.

**Supplementary Fig. S8.** Top five over-representation analysis results based on 34 MR significant proteins from UKB-PPP, using GO, WikiPathway, and DsigDB databases. Categories include BP, CC, MF, and DsigDB. BP and WikiPathways results are omitted due to a lack of statistically significant enrichment. Circle sizes represent the number of proteins enriched in each pathway or term. MDD, minimum detectable difference; DPA, differential protein abundance; GO, Gene Ontology; BP, biological process; CC, cellular component; MF, molecular function; DsigDB, Drug Signature Database.

**Supplementary Fig.** **S9.** Flowchart summarizing the overall study design. MR, mendelian randomisation; DPA, differential protein abundance; OA, osteoarthritis; UKB, UK Biobank; B-H, Benjamini–Hochberg; UKB-PPP, UK Biobank Pharma Proteomics Project; meta-GWAS, meta-analysis of genome-wide association studies; GO consortium 2.0, Genetics of Osteoarthritis Consortium 2.0; GO, Gene Ontology; BP, biological process; CC, cellular component; MF, molecular function; DSigDB, Drug Signature Database.
